# THE QUALITY OF NURSING CARE PROVIDED TO PATIENTS LIVING WITH DEMENTIA: FROM COMMUNICATION- TO FUNCTION-FOCUSED CARE

**DOI:** 10.1093/geroni/igad104.1107

**Published:** 2023-12-21

**Authors:** Barbara Resnick

## Abstract

Although shortages in nursing abound across all levels of care, it is important that the little time nurses have to engage with patients living with dementia while hospitalized be optimal. The Function Focused Care for Acute Care intervention helps nurses interact with patients living with dementia in a way that engages them in physical activity during all care interactions. This includes such things as walking a patient to the bathroom instead of using a commode chair or bedpan, role modeling for patients and having them wash their face and upper body, walking patients in the hallway when agitated, or doing hand over hand feeding and bathing if the patient is totally dependent. Function focused care provides staff with ways in which to interact with patients living with dementia to be more positive and encouraging with regard to activity and engagement versus neutral or negative. Positive interactions are beneficial in that they encourage participation and engagement in care, neutral interactions are brief and indifferent and provide no encouragement, and negative interactions restrict patients often due to inappropriate safety concerns or for the convenience of the health care team. This symposium will provide an overview of the way in which nurses provide function focused care to patients living with dementia when hospitalized, the quality of care interactions provided and the interventions used in our Function Focused Care work that have helped to improve the percentage of time function focused is provided and the quality of care interactions.

